# Earnings management, business strategy, and bankruptcy risk: evidence from Indonesia

**DOI:** 10.1016/j.heliyon.2020.e03317

**Published:** 2020-02-03

**Authors:** Dian Agustia, Nur Pratama Abdi Muhammad, Yani Permatasari

**Affiliations:** Faculty of Economics and Business, Universitas Airlangga, Indonesia

**Keywords:** Earnings management, Business strategy, Bankruptcy risk, Accounting, Industry management, Business management, Strategic management, Risk management, Business

## Abstract

The purpose of this study is to examine the effect of accrual earnings management and business strategy to bankruptcy risk. Multiple Least Square (MLS) regression and robust regression of M-Estimator regression are performed on financial data of 1,068 non-financial firms listed on the Indonesia Stock Exchange (IDX). The result indicates that there is no relationship between earnings management and bankruptcy risk, while firms that implement either one of two generic business strategies of cost leadership or differentiation, significantly mitigate the risk of bankruptcy. The effect of earnings management to bankruptcy risk is essential for external stakeholders, such as investors and creditors, to assess bankruptcy risk, financial capability, and credit worthiness of a firm, while business strategy effect on bankruptcy risk benefits internal stakeholders, such as managers, in formulating strategies to deal with going concern issues.

## 1 Introduction

Predicting, measuring, mitigating and assessing bankruptcy risk of a company has been a long-standing focus for investors prior to investing their capital. This is because investment provides means to achieve value maximization in terms of either capital gains or dividend payment. Nevertheless, maximization of value can only happen if capital providers selectively choose a profitable and sustainable business from which they can get the maximum portion of business income.

Bankruptcy risk by companies is a “hot topics” in management, business and accounting literature due to its implications for stakeholders' decisions (Lukason and Camacho-Miñano, 2019). Bankruptcy risk is risk for businesses being unable to meet their obligation, necessitating action through legal means of filing for bankruptcy to either reorganize their debt or liquidate their assets to meet such obligation (Bryan et al., 2013). Given that bankruptcy affects creditors, employees, managements, society, and shareholders, assessment of bankruptcy risk and factors that have an effect on bankruptcy risk is of major importance for businesses' stakeholders, in particular, shareholders.

One practice that obscures assessment of bankruptcy risk is earnings management. Earnings management occurs when management exert their influence to deliberately alter the truth and fairness of a financial statement with the purpose of either concealing real economic condition or attaining private gain out of contractual outcomes which rely on accounting numbers (Healy and Wahlen, 1999). Although earnings management does not belong to fraud, as it is still in accordance with the prevailing financial reporting standards of IFRS and GAAP (Stolowy and Breton, 2004) and is frequently used as a strategy in financial reporting to the extent that it is still capable of providing value-relevant information (Kwag and Stephens, 2009), when earnings management obscures investors' rational calculation, the devastating effect is undeniable since it can degrade the quality of information related to profits presented in the financial statements. The low quality of information contained in the financial statements will adversely affect the company's financial performance (Soewarno, 2018). In 2002, PT. Kimia Farma, Tbk, an Indonesian pharmaceutical state-owned company, received financial punishment after deliberately inflating its earnings as much as IDR 3.6 billion (Tempo, 2003). In September 2015, Toshiba Corporation was proven guilty of overstating its earnings as much as USD 2 billion over a seven-year accounting period, which, in turn, led to the resignation of its CEO and President of Toshiba Corporation, Hisao Tanaka, and inflicted financial loss on its investors (Addady, 2015).

In contrast to earnings management, which poses a threat to businesses' going concern ability, business strategy renders businesses more productive and profitable, mitigating bankruptcy risk in the future (Bryan et al., 2013). A business can implement two generic strategies of cost leadership and differentiation, or combine both strategies to withstand a turbulent and competitive business environment (Porter, 1980). Cost leadership is implemented through both cost efficiency (maximizing input to yield desirable output) and asset parsimony (maximizing fixed asset capacity to yield desirable output) (David et al., 2002; Hambrick, 1983). For example, Southwest Airline minimized its operational expense by almost exclusively using Boeing 737–800 and keeping employee retention rate at 92% (Chishty-Mujahid, 2017). Differentiation, on the other hand, is executed through concerted and relentless effort to pursue uniqueness of products and unparalleled brand loyalty (Bryan et al., 2013). For example, Garuda Indonesia, rated as a 5-star Indonesian Airline by Skytrax review in 2018, focuses on a relatively segmented market niche of middle to upper class passengers to develop brand loyalty by offering on-board entertainment and a priority passenger program (Al-Hafiz, 2016).

Despite previous studies conducted to investigate the relationship between earnings management and bankruptcy risk as well as business strategy and bankruptcy risk, the results remain inconsistent. Campa and Miñano (2013) investigate earnings manipulation behavior of 1,387 unlisted firms filing for bankruptcy in Madrid in 2010 and reveal that management inflate earnings upwards in extensive amounts for non-healthy firms, covering their poor performance. Tabassum et al. (2015) find the effect of earnings management to future performance using 119 firms listed on the Karachi Stock Exchange from 2004 to 2011. The result affirms that earnings management negatively influences future financial performance ratios. On the other hand, Charitou et al. (2007b) and Agrawal and Chatterjee (2015) suggest that healthy firms engage in higher earnings management and distressed firms tend to be more conservative about their financial condition. Moreover, in spite of previous studies conducted to discern earnings management, business strategy, and bankruptcy risk, the approaches are retrospective on whether bankrupted firms used to engage in earnings management. Lastly, this study acknowledges various methods of earnings management calculation as the research gap in this field of study.

This study investigate the relationship between earnings management, business strategy and bankruptcy risk. Samples used in this study are 1,068 firm-year observations of companies listed on the Indonesia Stock Exchange (IDX) for the period of 2014–2016. Earnings management is measured using discretionary accruals following the Modified Jones model by Dechow et al. (1995), while cost leadership and differentiation strategy are measured using asset turnover of operation and profit margin, respectively (Bryan et al., 2013; Hambrick, 1983; Wu et al., 2015). Bankruptcy risk is measured using Altman Z score following the model developed by Altman (1968). The result shows that earnings management has no relationship with bankruptcy risk, while business strategy has positive and significant effect towards bankruptcy risk.

This is the first study to assess earnings management, business strategy and bankruptcy risk. Moreover, this study provides contextualization of earnings management, business strategy and bankruptcy risk on the Indonesia business landscape, which has never been done in the previous literatures. Therefore this study provides important insights to both internal and external stakeholders of businesses. Firstly, it informs management of the effect of earnings management and the extent to which it influences financial performance and how significant business strategy may improve financial performance, avoiding greater bankruptcy risk in the future. Secondly, it provides applicable information to assist loan or investment decision for firms subject to earnings management and business strategy.

The rest of this study is organized as follows. In the next section, the literature review used in this study is being discussed. In the third section, the research methodology, proxy, and measurements of variables employed in this research were elaborated. In the fourth and fifth section, this study discuss the results and provide a conclusion to this study.

## 2 Literature review

### 2.1 Agency theory

According to Jensen and Meckling (1976), agency theory pertains to the contractual relationship of one or more persons, called the agent, to engage in a series of business processes or decision-making in order to perform a service on the principal's behalf, who delegates the authority of managing the business. In the agency relationship, there may be conflicts that occur due to differences in interests between the principal and the agent. Shareholders demand increased profitability and company dividends, while managers are agents who are motivated to maximize the fulfillment of economic and psychological needs (Soewarno, 2018). This contractual relationship is tightly associated with agency problems and agency costs. Agency problems elucidate that a contractual relationship may or may not be obeyed by both individuals due to asymmetric information in which one party receives or possesses more information than another, usually the agent. The first problem is that both parties are subject to their own utility maximization, resulting in the fact that the agent may not always act in the best interest of the principal. Second, asymmetric information occurs as agents have access and are closely tied to the daily operation of the business, putting the principal in the position of limited access of information of the true business performance (Gray and Manson, 2007).

In addition to agency problem, the occurrence of agency cost also pervades the principal-agent contractual relationship. Agency cost is cost incurred due to the principal-agent relationship in managing a business, which includes, (1) the monitoring expenditure by the principal, (2) the adherence cost by the agent, and (3) the residual loss. Agency cost applies to both principal and agent and may substantiate in the event where agency problem does take place.

### 2.2 Bankruptcy risk theory

Outecheva (2007) posits that there are two perspectives that arguably dominate the discussions upon the definition of bankruptcy risk, these are: (1) event-oriented definition of bankruptcy and (2) process-oriented definition of bankruptcy. Under event-oriented definition, bankruptcy is seen as a discrete event; depending on the first event to occur, bankruptcy can be seen as the time when a company files for bankruptcy, overdraws its bank account, or does not pay its preferred stock dividend (Beaver, 1966). In comparison, under the process-oriented definition introduced by Turetsky and McEwen (2001), bankruptcy is a series of sequential events that begins with firms experiencing reduction in cash flow up to experiencing negative cash flow, then dividend payout reduction, followed by bankruptcy filing. The process-oriented definition errs on the side of looking at bankruptcy risk as series of events that captures the spectrum of, not only legal ground of bankruptcy filing, but also the financial distress condition which may or may not lead towards bankruptcy filing.

In addition to the definition of bankruptcy as proposed by bankruptcy risk theory, the theory also identifies causes of financial distress which may lead to bankruptcy. Karels and Prakash (1987) identify two factors of financial distress; internal and external causes. Internal causes, or the endogenous factor, apply to specific firms and are reflected through poor management, earnings management practices and unprofitable projects. The external or exogenous factor is pervasive, it affects all firms systemically, including market risks and regulatory changes.

Bankruptcy risk is the whole spectrum of events and possibilities of business to experience financial distress which may or may not lead into bankruptcy filing (Altman, 1968). The proxy of bankruptcy risk used in this study is Altman-Z score, as introduced by Altman (1968). Altman's Z-score is a measuring tool that can be used to see the possibility of bankruptcy experienced by companies by combining profitability, leverage, liquidity, solvency, and activities (Cooper and Uzun, 2019). The reason as to why Altman-Z score is chosen as proxy is that it has been proven to be one of the most robust bankruptcy predictions model, having been used in numerous researches, including Ohlson (1980) and Zmijewski (1984). Z-score generated by the model indicates financial strength of a firm, the lower the Z-score, the greater bankruptcy risk it may experience.

(1) Zscore=1.2(WC)+1.4(RE)+3.3(EBIT)+0.6(MVE)+0.999(S)

where:

WC: working capital (current assets – current liabilities) scaled by total assets
RE: retained earnings scaled by total assets
EBIT: earnings before interests and taxes scaled by total assets
MVE: market value of equity scaled by total liabilities
S: sales scaled by total assets

### 2.3 Earnings management

Healy and Wahlen (1999) argue that earnings management is the act of management to exert influence on financial statements or structure transactions in such a way as to alter reported information in the financial statement for the purpose of either misleading shareholders about the underlying economic performance of the firm or to influence the contractual outcomes that rely heavily on accounting numbers. Earnings management refers to the use of such accounting practices that produce desired financial statements that reflect the financial position and financial performance of a healthy organization. This is done because sound financial statements can provide an overview of the stability and consistency that exists in the organization (Vishnani et al., 2019). Verbruggen et al. (2008) contend that management motives in employing earnings management, among many others, can be classified into (1) stock market incentives, (2) signaling or concealing private information, (3) political costs, (4) projecting CEOs' good performance, and (5) internal motives.

According to Joosten (2012), earnings management practices are classified as either accrual-based earnings management and real earnings management. Real earnings management (REM) is implemented through executing practices that deviate from normal business activities in terms of operating and investing activities, and financing activities. For example, extending project development to avoid project development expense under provision of IAS no.38 or buying outstanding shares in order to increase the level of earnings per share (Bens et al., 2003). Accrual-based earnings management (AEM) is implemented through managerial influence and discretion to accruals, which is also permitted by prevailing accounting standards and regulations. For instance, determination of fixed asset estimated useful life, salvage value, the depreciation method, asset impairment, and estimation of bad debt expense.

Several models have been developed in order to measure degree of earnings management, including Roychowdhury (2006) model of REM, Healy (1985), DeAngelo (1986), Jones (1991), and Dechow et al. (1995) model of AEM. Taking into account all advantages and disadvantages of each model as provided above, this study focuses on accrual-based earnings management by using the Modified Jones Model to measure earnings management activities. The reason as to why the Modified Jones Model is chosen is that it is a revised version of the Jones Model developed by Jones (1991) that provides more accurate results of discretionary accruals (Dechow et al., 1995).

Discretionary Accruals (DA) is used as proxy of earnings management in this study in which the model of measurement follows the Modified Jones Model as firstly introduced in Dechow et al. (1995). The following steps are used to measure Discretionary Accruals (DA) in this study.

(a) Step 1 Measurement of Total Accrual (TA)

Regression analysis is performed on total accruals items, net sales revenue, property, plant and equipment to obtain values for coefficients α_1,_ α_2,_ and α_3._

(2) $$\frac{TAC_{jt}}{A_{jt-1}}=\;\alpha 1\;\left(\frac{1}{A_{jt-1}}\right)+\;\alpha 2\;\left(\frac{\Delta REV_{jt}-\;\Delta REC_{jt}}{A_{jt-1}}\right)+\;\alpha 3\;\left(\frac{PPE_{jt}}{A_{jt-1}}\right)+\varepsilon_{jt}$$

(b) Step 2 Measurement of Non-Discretionary Accrual (NDA) or Expected Normal Accrual

After obtaining the values of coefficients α1, α2, and α3, these values are used to measure the Non-Discretionary Accruals for each firm by using the following formula.

(3) $$NDA_{it}=\;\alpha 1\;\left(\frac{1}{A_{it-1}}\right)+\;\alpha 2\;\left(\frac{\Delta REV_{it}-\;\Delta REC_{it}}{A_{it-1}}\right)+\;\alpha 3\;\left(\frac{PPE_{it}}{A_{it-1}}\right)+\;\varepsilon_{it}$$

where:

NDA it: Firm i's non-discretionary accruals in time period t
(c) Step 3 Measurement of Discretionary Accrual (DA)

Finally, the value of Discretionary Accrual is acquired by subtracting Non-Discretionary Accrual (NDA) to Total Accrual (TAC).

(4) $$\left|DA_{it}\right|=\;\frac{TAC_{it}}{A_{it-1}}-\;NDA_{it}$$

where:

DAit: Firm i's discretionary accruals in absolute value

### 2.4 Business strategy

Porter (1980) argues that business strategies are the policy and stance that a business entity takes in response to their competitive business environment and a set of values or product mix that they develop the aim of which is to outcompete competitors. While, Chen and Keung (2019) argues that business strategies can be characterized by how companies decide to compete, pursue, achieve, and maintain their competitive advantage in the industrial sector. Porter (1980) contends that there are three business strategies which a company may choose to employ, cost leadership, differentiation, and focus strategy. This study excludes focus strategy as it is derived from cost leadership and differentiation as strategy which is implemented on a specific market niche (Wu et al., 2015).

Cost leadership emphasizes on being the lowest cost producer in the industry at a given level of quality. The strategy is implemented through pursuit of economy of scale, TQM, JIT, and inventory management system using EOQ to minimize the cost of holding inventory. Differentiation strategy, on the other hand, focuses on creating value by generating high margins in pursuit of distinctive product features that separate it from competitors.

Two business strategies are employed in this study, cost leadership and differentiation. Cost leadership is a strategy used by cost leaders in order to achieve competitive advantage of price by minimizing cost through achieving operational excellence. Following previous research by Hambrick (1983) and David et al. (2002), Asset Turnover of Operation (ATO) is a critical measurement of cost leadership in which the higher the ratio between output and input, the better the firm utilizes its resources to achieve operational excellence and, thus, indicates the degree of cost leadership employed by the firm. We use asset turnover of operation (ATO) as proxy of cost leadership strategy and use the following equation according to Wu et al. (2015) to compute it.

(5) $$ATO=\;\frac{Operating\;sales}{Average\;operating\;assets}$$

where:

Operating assets = Total asset – cash – short term investment

Differentiation business strategy is a strategy that emphasizes on attaining competitive advantage through uniqueness and distinctive features of goods and service offered to customers. Profit margin is used as proxy for differentiation strategy in this research following research by Wu et al. (2015). Selling and Stickney (1989) argue that differentiation is tightly related with profit-margin maximizing strategy, where a business aims to maximize its profit by offering superior products. In addition, in order to make distinctive products. The business must put frantic efforts into constant product development through R&D expenditure. Therefore, profit margin is a proxy for differentiation strategy is and is measured using the following formula.

(6) $$PM=\frac{\left(Operating\;income+R\&D\;Exp\right)}{Sales}$$

### 2.5 Hypothesis development

#### 2.5.1 The effect of earnings management towards bankruptcy risk

Firms tend to report inflated-financial performance for various reasons, including to meet forecasting benchmark (Verbruggen et al., 2008), to meet earnings target (Haw et al., 2005), or to conceal poor financial condition (Rosner, 2003). For instance, earnings management is employed to avoid violations of debt covenant in times of financial distress, which allows greater access of leverage (Charitou et al., 2007a). The use of earnings management indicates management orientation towards short-term accomplishment (i.e. investors' confidence, positive managerial review, etc.) rather than long term-goal of accountability, and transparency which may accomplish sustainable stream of liability or equity investment and, thus, reduce firms' going concern ability. Furthermore, concealment of poor operational or financial performance through earnings management hinders early diagnosis to fix the problem, leaving the problems lurking within daily operations, thus making the firm unable to withstand a competitive environment. Leach and Newsom (2007) provide empirical evidence of positive discretionary accruals (DCA) for firms with impending bankruptcy cases, which affirms excessive use of earnings management as a major cause of firms filing for bankruptcy. Tabassum et al. (2015) affirm that there is a negative and significant relationship between earnings management and future financial performance indicators. Based on the discussion, the following hypothesis is proposed:

H1 Earnings management has positive effect towards bankruptcy risk.

#### 2.5.2 The effect of business strategy towards bankruptcy risk

Porter (1980) contends that businesses withstand competition in a competitive environment by using or implementing strategies different to those of their competitors in order to obtain competitive advantage, which, in turn, leads them to their business goals. Porter's framework of competitive strategy posits two generic business strategies, cost leadership and differentiation. Cost leadership primarily focus on productivity by employing cost efficiency (minimizing cost to produce certain level of output) and asset parsimony (optimizing the use of fixed asset to produce certain level of output). Differentiation in the other hand, revolves around developing product uniqueness, customer loyalty, and unique distribution channels with the aim of generating high margins.

Despite the way each strategy is executed, both strategies aim to outperform competitors, generate high level of productivity or yield the greatest profit margin, or, in other words, ensure business sustainability to withstand a competitive environment and minimize the risk of going out of business. This notion corresponds to research done by Bryan et al. (2013) that investigates the effect of business strategy towards bankruptcy risk and which provides empirical evidence that business strategies improve financial performance and, thus, mitigate the risk of bankruptcy. Hence, the second hypothesis is proposed.

H2 Business strategy has negative effect towards bankruptcy risk.

## 3 Methods

### 3.1 Data

This study use data from ORBIS database. Firms in financial industries were excluded in the sample due to requirement to adhere to stricter prevailing regulation and different accounting treatment and interpretation of bankruptcy risk (Fama and French, 1992). The dataset contains 1,068 firm-year observations that span from 2014 – 2016 that listed in Indonesia Stock Exchange (IDX). This research mainly uses hypothesis testing using Multiple Linear Regression analysis in order to execute research model and establish relationship between independent and dependent variable using the tool of Stata Corp. STATA MP Version 14.0.

### 3.2 Research empirical model

(7) Zscore=β0+β1DAit+β2ATOit+β3PMit+β4Leverage+β5Sizeit+β6Liquidityit+Lossit+Year&Industry+εit

This study employ the above research model to examine both H1 and H2. where Z score measures the level of Altman Z-score of firm i in period of t calculated based on the model developed by Altman (1968). The greater the value of Z-score, the greater the firm's financial strength. DA indicates the degree of earnings management employed by firm i in period of t as measured by Eq. (4). ATO and PM are proxies of cost leadership and differentiation, respectively, as measured by Eqs. (5) and (6). We expect β1 to be negative and significant, while β2 and β3 to be positive and significant in accordance to our hypotheses.

Apart from previously mentioned variables, several control variables were incorporated within the research model in accordance with the prior research (Agrawal and Chatterjee, 2015; Aharony et al., 2000; Bryan et al., 2013; Oktovianti and Agustia, 2012). Leverage (Leverage) is the use of debt from creditors in the firms' capital structure (Ross et al., 2000:23) and measured by total liabilities to asset ratio. We expect leverage to be negatively related to Z-score as degree of leverage significantly exposes firm to greater risk of bankruptcy or lower Z-score (Black and Scholes, 1973).

Firm's size (Size) is the relative size of firms in a given industry through a natural logarithm of its total assets (Wu et al., 2015). The greater the total assets of the firm, the greater its size, which corresponds to different behavior, financial condition and the regulatory requirements to which it should adhere. We expect firm size to have positive relationship with Z-score, as the greater the size of the firm, greater managerial competencies and prudential principal are upheld by board of directors (Theodossiou et al., 1996). Liquidity (Liquidity) is defined as the speed and ease of assets to be converted into cash (Ross et al., 2000:22). Liquidity is measured through cash holdings ratio, total cash and cash equivalent divided by firms' total assets. Loss (Loss) is the condition where firms experience negative net income in a given accounting period. In this study, it is designated to be the dummy variable assigned to companies which experience loss or negative net profit during the year in which value is assigned to be 1 if the firm experiences loss during the year, otherwise 0. We expect loss to have negative relationship with Z-score as negative profit indicates little to no earnings to be reinvested in the operation and financing activities, which deteriorates financial condition over time (Bryan et al., 2013).

## 4 Results and discussion

### 4.1 Descriptive statistics

Table 1 provides descriptive statistics of the dataset used within this study. The average value of Z-score is 3.27. Discretionary accruals (DA) averages 0.08, while the maximum and minimum value are 0.00 and 0.39, respectively. Asset turnover of operation (ATO) and profit margin (PM) as proxies of business strategy average 0.97 and 0.09, respectively. Leverage (Leverage) averages 0.53, which indicates that it comprises most of the capital structure of firms in the dataset. Size (Size) averages 21.68 with maximum and minimum value of 25.20 and 17.99. Liquidity (Liquidity) and Loss (Loss) average 0.09 and 0.27, respectively.

**Table 1:** Descriptive statistics.

Variable	N	Minimum	Median	Maximum	Mean	Std. Deviation
Z-score	1068	-9.98	2.46	22.51	3.27	4.06
DA	1068	0.00	0.06	0.39	0.08	0.07
ATO	1068	0.05	0.78	3.97	0.97	0.80
PM	1068	-0.98	0.77	0.78	0.09	0.23
Leverage	1068	0.07	0.49	3.03	0.53	0.38
Size	1068	17.99	21.66	25.20	21.68	1.56
Liquidity	1068	0.00	0.06	0.46	0.09	0.10
Loss	1068	0.00	0.00	1.00	0.27	0.44

*Source*: Data processed using STATA, 2018.

### 4.2 Multicollinearity test

Table 2 provides the result of the multicollinearity test. As shown by the table, all variables' VIF value are located between f 1 and 10, with the highest VIF value of 7.56 belonging to one of the categorical variables of industry, and the minimum value of 1.15 belonging to discretionary accruals. Overall, the mean value of VIF is 2.44, which proves that the dataset is not subject to multicollinearity.

**Table 2:** Multicollinearity test result.

Variables	VIF	1/VIF
DA	1.15	0.871485
ATO	1.65	0.606405
PM	1.77	0.565789
Leverage	1.30	0.772063
Size	1.19	0.840791
Liquidity	1.24	0.803640
Loss	1.80	0.555215
Year		
Year 2014	1.41	0.707702
Year 2015	1.38	0.724720
Industry		
ind1	2.04	0.491254
ind2	2.41	0.415014
ind3	7.56	0.132286
ind4	3.15	0.317317
ind5	3.40	0.294085
ind6	2.29	0.437110
ind7	3.22	0.310465
ind8	4.57	0.218778
**Mean VIF**	**2.44**	

*Source*: Data processed using STATA, 2018.

### 4.3 Pearson correlation

Pearson product moment or correlation, symbolized as *r,* is a widely known correlation coefficient and has been used in various journals to summarize the relationship between two variables that are straight-line or linear (Hauke and Kossowski, 2011). Table 3 depicts the relationship between dependent variable and independent variable as well as among variables within this study.

**Table 3:** Pearson correlation test result.

	Z-score	DA	ATO	PM	Leverage	Size	Liquidity	Loss
Z-score	1.000							
DA	-0.157*** (0.000)	1.000						
ATO	0.241***	-0.048	1.000					
	(0.000)	(0.115)						
PM	0.343***	-0.147***	-0.033	1.000				
	(0.000)	(0.000)	(0.287)					
Leverage	-0.486***	0.217***	0.102***	-0.252***	1.000			
	(0.000)	(0.000)	(0.001)	(0.000)				
Size	0.079***	-0.027	-0.116***	0.278***	-0.023	1.000		
	(0.010)	(0.377)	(0.000)	(0.000)	(0.457)			
Liquidity	0.296***	-0.090***	0.259***	0.131***	-0.246***	0.013	1.000	
	(0.000)	(0.003)	(0.000)	(0.000)	(0.000)	(0.682)		
Loss	-0.317***	0.136***	-0.264***	-0.567***	0.281***	-0.142***	-0.179***	1.000
	(0.000)	(0.000)	(0.000)	(0.000)	(0.000)	(0.000)	(0.000)	

*Notes: P-values* in parentheses *P < 0.1, **P < 0.05, ***P < 0.01.

*Source*: Data processed using STATA, 2018.

Z-score has correlation coefficient of -0.157 and is significant at the level of 1% with DA, indicating that Z-score is inversely related with DA. Moreover, Z-score, ATO and PM are shown to have positive relationship with coefficient of 0.241 and 0.343, respectively, significant at 1%. With regard to control variables, Leverage and Loss impose negative influence on Z-score, with -0.486 and -0.317 of correlation coefficient, respectively. Meanwhile, Size and Liquidity have 0.079 and 0.296 correlation coefficients, respectively. Based on the information presented in Table 3, Z-score has significant relationship to all of the independent variables. Hence, it can be inferred that as the level of discretionary accruals increases or companies engage in higher earnings management, both upwards and downwards earnings management, the level of financial strength measured by Z-score decreases. In other words, firms which are actively engaging in earnings management may experience higher bankruptcy risk in the future. This finding corresponds with previous research done by Tabassum et al. (2015), and Campa and Miñano (2013) which demonstrate firms experience unfavorable financial performance, as indicated by ROA, ROE, PE ratio and EPS, subsequent to undergoing earnings management.

### 4.4 Multiple regression result

Table 4 displays the result of Multiple Least Square (MLS) regression. As explained earlier, the result above has been adjusted for year and industry fixed effect, as well as Petersen (2009) cluster of firms and year fixed-effect to control for heteroscedacity and autocorrelation and to ensure that the output and standard error are robust of changes of condition. To control for normality distribution problem, the data are winsorized at 1%.

**Table 4:** Multiple regression result.

Variables	MLS Regression
DA	0.298 (0.19)
ATO	1.290*** (7.40)
PM	4.748*** (6.82)
Leverage	-4.701*** (-12.60)
Size	0.113 (1.53)
Liquidity	3.841** (2.52)
Loss	0.347 (1.27)
Constant	1.297 (0.76)
Year and industry Dummies	Included
F	29.59
R^2^	0.392
R-mse	3.194
*N*	1068

*Notes: t* statistics in parantheses *t > 1.65, **t > 1.96, ***t > 2.58.

Dependent variable is Z-score measured using Altman-Z score.

*Source:* Data processed using STATA, 2018.

Looking at the table above, the coefficient of DA is 0.298, and not significant. The result is contradictive towards the hypothesis and corresponds to previous research which addresses a positive relationship between DA and Z-score (Agrawal and Chatterjee, 2015; Aharony et al., 2000; Chen et al., 2010). The results of independent variable of ATO and PM are consistent with the hypothesis and previous researches. ATO coefficient is positive and significant (coefficient = 1.290; *t*-statistics = 7.40). This indicates that firms which implement cost leadership strategy through which the main objective is to become the lowest cost producer in the industry experience significant improvements in financial performance. Moreover, the result is also indicative that firms that implement differentiation strategy significantly experience lower bankruptcy risk. This can be seen by the coefficient of PM which is also positive and significant (coefficient = 4.748; *t*-statistics = 6.82). This result indicates that firms that implement either of the generic business strategies of cost leadership or differentiation yield better financial strength and are, thus, less likely to bear significant bankruptcy risk.

Moreover, Z-score, ATO and PM are shown to have positive relationship with coefficient of 0.241 and 0.343 respectively, significant at 1%. The result indicates that business strategy amplifies firm's financial position, or in the other words mitigate the risk of bankruptcy. This findings is consistent with previous study done by Bryan et al. (2013), which proves firms that select either cost leadership or differentiation strategy as part of their business strategy experience better financial performance.

With regard to control variables, firm's leverage (Leverage) and liquidity (Liquidity) are proven to be significant determinants of bankruptcy risk. Firm's leverage is proven to have negative and significant relationship with Z-score (coefficient = -4.701; *t*-statistics = -12.60). This indicates that additional leverage significantly exposes firms to higher risk of bankruptcy since it renders them to be very cautious on using either short-term or long-term debt due to the obligation of paying interest as the cost of borrowing the money, where greater debt means more interest to be paid in the future (Black and Scholes, 1973). As for liquidity (Liquidity), it is proven to have positive and significant relationship (coefficients = 3.841; *t*-statistics = 2.52). Moreover, firms' relative size (Size) and loss indicator (Loss) are proven to have positive and insignificant results with coefficients of 0.113 and 0.347, respectively.

### 4.5 Robustness test

Being fully aware of the vulnerability of Multiple Least Square (MLS) regression and Pearson's correlation, which is susceptible to presence of outliers which significantly distort the result, an additional robustness test is employed, the main purpose of which is to ensure resistant result in the presence of outliers (Mukaka, 2012; Pernet et al., 2013). Robust regression provides assurance of results by minimizing outliers' effect (Alma, 2011). Among robust regression methods, the M-Estimator regression by Huber (1964) is used within this study. The reasons are, first, M-Estimator provides greater than 64% of Gaussian error distribution while keeping robustness of the model. Secondly, it works by minimizing the function of ῤ of errors, rather than minimizing sum of squared errors, protecting the data against vertical outliers. Thirdly, it has breakdown points up to 25% of outliers and 15% of leverage points (Alma, 2011). This function is available in STATA through *rreg* command (Verardi and Croux, 2008).

Table 5 presents the comparison between MLS regression and M-Estimator regression. Both regressions have been adjusted for year and industry fixed effect, as well as the Petersen (2009) cluster to control for heteroscedacity and autocorrelation. Thus, the regression is robust to year-industry fixed effect, heteroscedacity and autocorrelation.

**Table 5:** MLS regression and M-Estimator regression result.

Variables	MLS Regression	M-Estimator Regression
DA	0.298	0.0187
	(0.19)	(0.04)
ATO	1.290***	1.142***
	(7.40)	(21.26)
PM	4.748***	1.801***
	(6.82)	(9.19)
Leverage	-4.701***	-4.071***
	(-12.60)	(-40.67)
Size	0.113	0.132***
	(1.53)	(5.66)
Liquidity	3.841**	-0.636
	(2.52)	(-1.64)
Loss	0.347	-0.315***
	(1.27)	(-3.11)
Constant	1.297	0.959*
	(0.76)	(1.72)
Year and Industry Dummies	Included	Included
F	29.59	195.7
R^2^	0.392	0.760
R-mse	3.194	1.089
*N*	1068	1068

*Notes: t* statistics in parentheses *t > 1.65, **t > 1.96, ***t > 2.58.

Dependent variable is Z-score measured using Altman-Z Score.

*Source:* Data processed using STATA, 2018.

DA remains insignificant under MLS regression and robust regression, whereas ATO and PM are proven to have positive and significant results under both regression models (MEstimator coefficient = 1.142; M-Estimator *t*-statistics = 21.26; MLS coefficient = 1.290; MLS *t*-statistics = 7.40). The result indicates that ATO and PM's effects towards bankruptcy risk are robust, as, given two models of regression, it remains positive and significant. The positive and significant result of ATO and PM suggests that companies which pursue either cost leadership strategy or differentiation strategy mitigate their bankruptcy risk. Hence, business strategy is a mitigating factor of bankruptcy risk.

As for the control variable of leverage (Leverage), it is proven to have significant and negative relationship to bankruptcy risk under both regressions, MLS and M-Estimator regression (M-Estimator coefficient equals to MLS coefficient = -4.071; M-Estimator *t*-statistics = -40.67; MLS *t*-statistics = 12.60). In contrast to the MLS regression result, firms' relative size (Size) and liquidity (Liquidity) exhibit a different result as it indicates a positive and significant relationship to bankruptcy risk (M-Estimator coefficient = 0.132; M-Estimator t statistics = 5.66; MLS coefficient = 0.113; MLS *t*-statistics = 1.53). Reasons behind the positive and significant relationship between firms' relative size and bankruptcy risk are improvements of managerial capacity, developments of business strategy and seamless cash flow over the duration of the business. This finding supports previous research (Chava and Jarrow, 2004; Lennox, 1999; Theodossiou et al., 1996) which found greater number of firms file for bankruptcy in the first five years of operation. As for liquidity (Liquidity), M-Estimator regression shows negative and insignificant relationship to bankruptcy risk (M-Estimator coefficient = -0.636; M-Estimator *t*-statistic = -1.64; MLS coefficient = 3.841; MLS *t*-statistics = 2.52). Categorical variable of loss indicator (Loss) also exhibits a different result from MLS regression, as it shows a negative and significant relationship whereas it shows a positive and insignificant result (M-Estimator coefficient = -0.315; M-Estimator *t*-statistics = -3.11; MLS coefficient = 0.347; MLS *t*-statistics = 1.27).

### 4.6 Discussion

#### 4.6.1 The effect of earnings management towards bankruptcy risk

The first hypothesis of this study which stipulates earnings management has positive effect on bankruptcy risk is rejected as research findings suggest insignificant effect at significance level (α) of 5% or *p*-value > α (M-Estimator *p*-value = 0.968). Referring to the dataset used within this study, it is evident that firms which engage in high level of earnings management do not exhibit poor financial performance. For instance, PT Delta Djakarta Tbk (DLTA) yield above-average financial performance despite high level of earnings management in 2014 (Z-score = 18.6175; DA = 0.1505). Another example, PT. Hanjaya Mandala Sampoerna Tbk (HMSP), which is considered as the second-highest firm with earnings management level, held the best financial performance (Z-score = 22.5106; DA = 0.3668) in 2015.

This result is in accordance to previous research done by Agrawal and Chatterjee (2015), which suggests that higher performing firms engage in higher earnings management, while distressed firms engage in lower earnings management and tend to reveal their true condition. It indicates that, contrary to the claim that earnings management is used as a means to disguise poor financial condition, it is used regardless of the financial condition of the firm. In addition, the use of earnings management may not always be related to the financial condition of the firm. The nature of the industry in which the firm operates also determines the likelihood as to whether firms engage in earnings management. According to previous study by Aharony et al. (2000), it is evident that firms which operate in less regulated industries, tend to have greater discretionary accruals.

Moreover, the result corresponds to agency cost in agency theory which mitigate the likelihood of management to divert from principals' interest (agency problem) since effort to hide poor financial condition by engaging into excessive earnings management can't be exercised due to layer of mechanisms such as audit opinion or other mechanism which are established to protect principals from inflated financial statements. This notion is supported by empirical evidence that most of the firms in the study which suffer lost in the period do not exhibit high level of earnings management. Furthermore, this result corresponds to previous study done by Chen et al. (2010) and Charitou et al. (2007a) which stipulates that bankruptcy-filing firms tend to be more conservative about their financial condition as they are subject to public and government attention and thus avoid engaging into excessive earnings management.

#### 4.6.2 The effect of business strategy towards bankruptcy risk

The second hypothesis posits that business strategy has a negative effect towards bankruptcy risk is accepted as data analysis techniques affirm that Business Strategy has positive and significant effect to financial strength at 5% level of significance (ATO & PM M-Estimator *p*-value = 0.0001). This indicates that firms which implement either cost leadership or differentiation strategy significantly experience better financial performance, which mitigates bankruptcy risk.

This result validates the theoretical framework by Porter (2008) with regards to the business strategy firms implement as a means of gaining competitive advantage and withstanding competitive environment within their respective industries. Although, the implementation may be different and firms may choose to implement either cost leadership or differentiation strategy, the framework suggests that firms that implement either of the business strategies perform better than their competition and, thus, carry lower bankruptcy risk.

Studies by Chang et al. (2012) and Bryan et al. (2013) validate the result of this study as both studies find that firm strategy, cost leadership and differentiation mitigate the risk of bankruptcy. Bryan et al. (2013) argued that firms which are successfully implement two generic Porter's strategies, have their bankruptcy risk reduces, since it will enable firms to have competitive advantage over their competitors. Although the implementation of the two strategies will be different, with cost leadership relying on productivity enhancements, while differentiation seeks innovation and brand loyalty, successful implementation of either strategy will lead to better performance. While Chang et al. (2012) find a positive link between cost leadership and productivity implying that as the level of cost leadership increases, the productivity also increases, firms will have better performance, hence bankruptcy risk will be mitigated.

Being the first study that puts spotlight on the link among earnings management, business strategy, and bankruptcy risk, this study shows that the effect of earnings management to bankruptcy risk are essential for external stakeholders such as investors and creditors to assess bankruptcy risk, financial capability, and credit worthiness of firm, while business strategy effect on bankruptcy risk benefits internal stakeholders such as managers in formulating strategies to deal with going concern issues.

## 5 Conclusions

Conclusions of this study with regards to the effect of earnings management and business strategy towards bankruptcy risk are two-folds. First, there is no relationship between earnings management and bankruptcy risk. In addition, the research corresponds to research done by Agrawal and Chatterjee (2015), Wu et al. (2015), and Aharony et al. (2000) which demonstrate that earnings management does not expose firms to greater risk of bankruptcy. Second, there is a negative and significant relationship between business strategy and bankruptcy risk. Businesses that implement either of the two generic strategies of cost leadership or differentiation significantly have better financial performance and, thus, lower risk of bankruptcy. These findings correspond to previous study by Bryan et al. (2013) that suggests business strategy mitigates bankruptcy risk.

This study is crucial for external stakeholders, such as investors and creditors, to assess the bankruptcy risk, financial capability and credit worthiness of firm, while business strategy effect on bankruptcy risk benefits internal stakeholders, such as managers, in formulating strategies to deal with going concern issues, therefore this research provide empirical evidence towards the use of business strategy as mitigating factor of bankruptcy risk.

### 5.1 Research limitation

Limitations within this research are as follows;

1. Although M-Estimator is employed as robustness regression and protects the result against vertical outliers, the model still prone to the effect of bad leverage points. Given abnormality of data used within this research, even after several data treatment to address data abnormality, it is inevitable to say that outliers, either vertical, good leverage, or bad leverage remain undetected.
2. Understanding that the period covered within this study only spans for 3 years, from 2014 to 2016, the statistical inference and generalization of the conclusion may only apply within this period.

### 5.2 Future research suggestion

Further research suggestions include employment of another model of robust linear regression that has a significantly greater Gaussian error efficiency and greater breakdown points which are capable of providing more bulletproof results against the effect of outliers, and expansion of the period of the study to obtain greater numbers of sample to obtain better statistical inference that represents broader areas of the whole population.

## Declarations

### Author contribution statement

Dian Agustia, Nur Pratama Abdi Muhammad, Yani Permatasari: Conceived and designed the experiments; Performed the experiments; Analyzed and interpreted the data; Contributed reagents, materials, analysis tools or data; Wrote the paper.

### Funding statement

This research did not receive any specific grant from funding agencies in the public, commercial, or not-for-profit sectors.

### Competing interest statement

The authors declare no conflict of interest.

### Additional information

No additional information is available for this paper.
